# The complex landscape of immune dysregulation in multisystem inflammatory syndrome in children with COVID-19

**DOI:** 10.1093/lifemedi/lnae034

**Published:** 2024-09-13

**Authors:** Jing Guo, Lie Wang

**Affiliations:** Institute of Immunology and Bone Marrow Transplantation Center, The First Affiliated Hospital, Zhejiang University School of Medicine, Hangzhou 311100, China; Department of Microbiology and Immunology, Stanford University School of Medicine, Stanford, CA 94305, USA; Institute of Immunology and Bone Marrow Transplantation Center, The First Affiliated Hospital, Zhejiang University School of Medicine, Hangzhou 311100, China; Liangzhu Laboratory, Zhejiang University Medical Center, Hangzhou 311100, China

**Keywords:** SARS-CoV-2, multisystem inflammatory syndrome in children, immune dysregulation, superantigen, autoantibody

## Abstract

The immune responses following SARS-CoV-2 infection in children are still under investigation. While coronavirus disease 2019 (COVID-19) is usually mild in the paediatric population, some children develop severe clinical manifestations or multisystem inflammatory syndrome in children (MIS-C) after infection. MIS-C, typically emerging 2–6 weeks after SARS-CoV-2 exposure, is characterized by a hyperinflammatory response affecting multiple organs. This review aims to explore the complex landscape of immune dysregulation in MIS-C, focusing on innate, T cell-, and B cell-mediated immunity, and discusses the role of SARS-CoV-2 spike protein as a superantigen in MIS-C pathophysiology. Understanding these mechanisms is crucial for improving the management and outcomes for affected children.

## Introduction

Severe acute respiratory syndrome coronavirus 2 (SARS-CoV-2), the virus responsible for the global COVID-19 pandemic, primarily spreads through respiratory droplets and can infect the respiratory system, leading to a spectrum of illnesses ranging from mild respiratory symptoms to severe pneumonia and acute respiratory distress syndrome (ARDS) [1]. Due to its rapid transmission and high infectivity, SARS-CoV-2 has caused a profound global health crisis, with significant disruptions to healthcare systems, economies, and daily life [2]. While the disease can be severe or even fatal, particularly among older adults and those with underlying health conditions, it generally has a milder impact on children [3–5]. Most children with COVID-19 are asymptomatic or experience mild symptoms. However, a rare but severe condition called multisystem inflammatory syndrome in children (MIS-C) has been reported in children following exposure to SARS-CoV-2 [6–9], which is a significant concern within paediatric healthcare. MIS-C typically occurs approximately 2–6 weeks after SARS-CoV-2 infection, characterized by a hyperinflammatory response that affects multiple organs, including the heart, lungs, kidneys, and gastrointestinal tract [10, 11]. The emergence of MIS-C has prompted increased research into unique immune responses in children and their roles in driving this severe inflammatory syndrome.

This manuscript aims to explore the complex landscape of immune dysregulation in MIS-C. We focus on the various immune mechanisms that underpin the syndrome and their clinical consequences. By integrating the underlying immune responses, we aim to offer a comprehensive overview of the pathophysiology of MIS-C, discuss current diagnostic challenges, and highlight effective treatment strategies. Through this exploration, we hope to contribute to the ongoing efforts to better understand and manage MIS-C, ultimately improving outcomes for affected children.

## The definition and incidence of MIS-C

In April 2020, cases of a novel hyperinflammatory disorder associated with COVID-19 affecting children and adolescents were first identified in the UK and Italy [8, 9]. These patients exhibited severe symptoms similar to Kawasaki disease (KD), resulting in approximately a 30-fold increase in such cases. Affected children showed symptoms such as persistent fever, abdominal pain, skin rashes, and cardiac involvement. Following the initial reports of this disorder, known as MIS-C, other countries quickly identified similar cases associated with SARS-CoV-2 infection [6, 7, 12–14]. In the initial phase of the pandemic, several terms were introduced to define this condition, such as paediatric inflammatory multisystem syndrome temporally associated with SARS-CoV-2 (PIMS-TS) and multisystem inflammatory disorder in children and adolescents [15]. Both the Center for Disease Control and Prevention (CDC) and the World Health Organization (WHO) have established case definitions for this syndrome, which was eventually named MIS-C. To improve specificity and reduce misclassification with other paediatric inflammatory conditions, the Council of State and Territorial Epidemiologists (CSTE) and CDC updated the surveillance case definition for MIS-C (Box 1), effective 1 January 2023 [10].

Box 1. **CSTE/CDC MIS-C surveillance case definition [10]**

- **Age**: < 21 years old, **Fever**: ≥ 38°C, **C-reactive protein**: ≥ 3.0 mg/dL.
- **Hospitalization**: Clinical severity requiring hospitalization or resulting in death.
- **SARS-CoV-2 infection or exposure:** Detection of SARS-CoV-2 RNA in a clinical specimen up to 60 days before or during hospitalization, or in a post-mortem specimen using a diagnostic molecular amplification test (e.g. PCR)
- **or** detection of SARS-CoV-2-specific antigen in a clinical specimen up to 60 before or during hospitalization, or in a post-mortem specimen
- **or** detection of SARS-CoV-2-specific antibodies in serum, plasma, or whole blood associated with current illness resulting in or during hospitalization
- **or** close contact with a confirmed or probable case of COVID-19 in the 60 days before hospitalization.
- **Multisystem involvement:** at least 2 of the following categories:
  – Cardiac iinvolvement indicated by left ventricular ejection fraction < 55%, **or** coronary artery dilatation, aneurysm, or ectasia, **or** troponin elevated to greater than laboratory normal range, or indicated as elevated in a clinical note.
  – Mucocutaneous involvement indicated by rash, **or** inflammation of the oral mucosa (e.g. mucosal erythema or swelling, drying, or fissuring of the lips, strawberry tongue), **or** conjunctivitis or conjunctival injection (redness of the eyes), **or** extremity findings (e.g. erythema or oedema of the hands or feet).
  – Shock.
  – Gastrointestinal involvement indicated by abdominal pain, **or** vomiting, **or** diarrhoea.
  – Haematologic involvement indicated by platelet count < 150,000 cells/μL, **or** absolute lymphocyte count < 1000 cells/μL.

**Figure 1.**
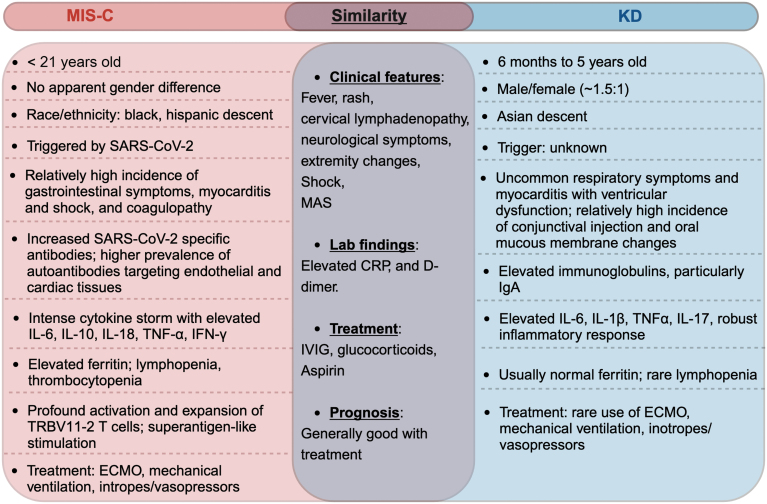
**The comparison of MIS-C and KD.**The similarities and differences in the epidemiological, clinical, and immunological features, treatments, and prognosis between MIS-C and KD are presented. Abbreviations: ECMO, extracorporeal membrane oxygenation; MAS, macrophagic activation syndrome; IVIG, intravenous immunoglobulin; CRP, C-reactive protein. This figure was created with BioRender.com.

MIS-C has been identified as a rare complication associated with SARS-CoV-2 infection in children under 21 years old (Fig. 1). While the median age of MIS-C patients is approximately 9 years, cases have been reported in infants as young as 2 weeks old. The symptoms of MIS-C appear to differ by age, with younger children more frequently presenting with mucocutaneous and gastrointestinal manifestations, whereas respiratory symptoms are more commonly observed in adolescents [16]. Infants appear to have a milder course of MIS-C than older children, with resolution of their illness after hospital discharge [17]. However, several risk factors including age under 1 year, obesity, and pre-existing conditions such as chronic cardiac and respiratory diseases, are often correlated with more severe clinical outcomes in MIS-C patients [18]. Abrams et al. reported that an age over 5 years was strongly associated with worse outcomes in MIS-C [19]. More ICU admissions were observed in MIS-C patients over 5 years. MIS-C incidence does not show apparent gender difference, though some cases showed a slight male predominance [20]. However, a stronger immune response has been observed in male MIS-C patients, characterized by higher levels of pro-inflammatory cytokines, chemokines, acute phase proteins (α-2M and CRP), growth factors (VEGF and TGFα), microbial translocation markers (iFABP, LBP, and EndoCAb), complement component (C1q, MBL and C3), and matrix metalloproteinases (MMP-8 and MMP-9) than in female MIS-C patients [21].

Cases of MIS-C have been reported worldwide, but there are significant variations in incidence across different regions, races, and SARS-CoV-2 variants. In the USA, the incidence during the first wave was estimated at 5.1 cases per million person-months or 316 cases per million SARS-CoV-2 infections in individuals under 21 years old [22]. Data from the USA, Europe, Asia, and Africa indicate a lower incidence of MIS-C during the periods dominated by Delta and Omicron variants [5, 23–26]. The epidemiology of MIS-C appears to have changed due to widespread exposure of the paediatric population to COVID-19 and vaccination efforts. Most cases are reported from European and American countries, while they are infrequently observed in Asian countries [27]. This disparity may be due to differences in infection rates among children, host factors, early treatment with immunomodulators, and genetic and ethnic differences [27, 28]. Notably, there is an over-representation of Black and Hispanic racial distribution among MIS-C patients (Fig. 1), which could be attributed to genetic and/or socioeconomic disparities [20, 29, 30].

The clinical severity and incidence of MIS-C have decreased with the emergence of subsequent SARS-CoV-2 variants. This reduction is likely due to a combination of factors, including increased natural and vaccine-induced immunity. Studies have shown that decreased MIS-C incidence is associated with COVID-19 vaccination efforts, especially when two doses are given [31–34]. Most hospitalized MIS-C patients eligible for vaccination were unvaccinated [31, 32]. Vaccination with two doses of BNT162b2 is associated with a significantly reduced likelihood of MIS-C in children ages 5–18 years [31].

## The comparison of MIS-C and KD

MIS-C was first recognized as a disease similar to KD, a rare but serious paediatric condition characterized by systemic vasculitis, which can lead to complications such as Kawasaki disease shock syndrome (KDSS) and macrophage activation syndrome [35, 36]. It was first described in 1967 in Japan by Dr. Tomisaku Kawasaki [36]. Despite some overlapping features, MIS-C and KD have distinct epidemiological, clinical, and immunological profiles (Fig. 1). Both conditions are linked to immune system alteration, systemic inflammation, and cytokine storms, but MIS-C elicits a more intense immune response, placing it further along the severity spectrum compared to KD [35].

While MIS-C is directly linked to SARS-CoV-2 infection, the exact cause of KD remains uncertain. Research suggests that KD is an immune-mediated condition triggered by infections in genetically predisposed individuals [35, 37–41]. KD typically affects children between 6 months and 5 years old, showing a male predominance of about 1.5:1 [35, 42]. The highest incidence rates are reported in Japan, China, and South Korea [35, 43]. In contrast, MIS-C cases are rarely reported in Japan and East Asian countries [14, 28, 44]. In the USA and Europe, MIS-C is more common in children of African and Hispanic heritage [22, 45].

Immunohistochemistry analyses have detected IgA plasma cells in inflamed tissues of KD patients, suggesting an antigen-driven immune response, although no single organism has been definitively linked to KD [35, 46]. Paediatric patients with MIS-C show strong immunoglobulin G (IgG) but weak IgM antibody responses to SARS-CoV-2 viral proteins [47–52], indicating its emergence weeks after viral exposure. The SARS-CoV-2 spike (S) protein might act as a superantigen, triggering a cytokine storm that leads to the toxic shock syndrome (TSS)-like presentation of MIS-C [5, 53–55]. Conversely, KD is believed to result from T cell activation by a conventional antigen [35, 56, 57]. Cytokine profiles differ between MIS-C and KD. MIS-C shows a robust increase in TNFα, IFNγ, and IL-10 production compared to KD [58]. The inflammatory mediator IL-17 is more prominent in KD than in MIS-C [59], while patients with severe MIS-C exhibit higher levels of circulating IFN compared to those with KD [58].

First-line treatments of KD include intravenous immunoglobulin (IVIG) and acetylsalicylic acid (aspirin), which typically lead to rapid improvement in symptoms [60]. In high-risk patients or those unresponsive to IVIG, steroids may be used to prevent coronary artery abnormalities. Other options for IVIG-resistant KD include infliximab (a monoclonal antibody to TNF), ciclosporin (a calcineurin inhibitor), and anakinra (an IL-1 receptor antagonist) [60]. Fewer than 10% of KD cases manifest as KDSS, requiring intravascular fluid resuscitation and vasoactive medication [35, 61]. Treatment for MIS-C aims to reduce the exaggerated inflammatory response, primarily using IVIG, glucocorticoids, and immunotherapy [62–65]. Most studies report that 70%–100% of MIS-C patients are treated with IVIG as the first-line agent, with satisfactory results. Steroids are the second most common treatment employed for MIS-C patients. Supportive care is crucial, including fluid resuscitation to manage shock, inotropic support for cardiac dysfunction or severe shock, and respiratory support through oxygen therapy or mechanical ventilation as needed.

## MIS-C and severe COVID-19 in adults

MIS-C and severe COVID-19 in adults share several characteristics, including fever, hyperinflammation, and hypercoagulability. Vella et al. reported that the immune landscape of patients with MIS-C overlapped with that of severely ill adults [66]. However, further investigation is needed to explore the unique immune responses in MIS-C. As shown in Fig. 1, the incidence of MIS-C does not show an apparent gender difference, while significantly more adult males are affected by severe COVID-19. No ethnic differences have been found in adults with severe COVID-19. The two conditions have distinct spatiotemporal profiles. In adults, severe COVID-19 is characterized by an early acute hyperinflammatory response to SARS-CoV-2 infection, typically emerging within 1 week after viral exposure. The severity of this condition is affected by several immune-modulating risk factors, including older age, underlying chronic diseases, immunosuppression, and pregnancy. Severe COVID-19 in adults predominantly affects the lungs, leading to pneumonia, significant pulmonary damage, respiratory distress, and subsequent systemic complications. In contrast, MIS-C represents a delayed hyperinflammatory response to SARS-CoV-2 infection. MIS-C patients usually do not develop severe respiratory illness but experience prominent cardiovascular, gastrointestinal, and hematological perturbations, along with other diffuse systemic manifestations and an autoimmune-like immunopathological signature [67, 68].

Multisystem inflammation syndrome is also reported in adults (MIS-A), which is a post-infectious hyperinflammatory disorder characterized by multi-organ failure occurring 2–6 weeks after SARS-CoV-2 infection. The majority of the patients are males (approximately 70%) with a mean age of 31.67 ± 10.02 years. The most common symptoms are fever and skin rash. The cardiovascular system was most frequently involved, followed by the gastrointestinal system and mucocutaneous involvement. The median hospital stay is around 8 days. The treatment and management of MIS-A patients are similar to those for MIS-C. During the hospital stay, 5%–7% of patients died from the illness [69, 70].

## Immune dysregulation in MIS-C: pathophysiology

Generally, MIS-C shares immune cell signatures and inflammatory parameters more closely with adults experiencing moderate-to-severe COVID-19, rather than with paediatric COVID-19 (pCOVID-19), which is mostly mild or asymptomatic. Immune activation in MIS-C is transient and decreases during recovery [48, 66, 71].

MIS-C is marked by abnormal blood cell counts, elevated inflammatory markers, and disturbances in pro-inflammatory mediators. Common findings include lymphopenia, neutrophilia, elevated levels of C-reactive protein (CRP), erythrocyte sedimentation rate (ESR), D-dimers, procalcitonin, fibrinogen, and ferritin, all correlating with disease severity [5, 71]. MIS-C patients admitted to paediatric intensive care unit (PICU) presented with relatively higher inflammatory markers, including CRP, ferritin, procalcitonin, and D-dimers. Additionally, higher IL-6 levels and more notable myocardial dysfunction were observed in MIS-C PICU patients [72]. Patients with MIS-C also have a higher rate of thrombotic events than those with mild to moderate symptoms, with the complement system possibly playing a role [73]. Elevation of levels of pro-inflammatory cytokines such as IL-6, IL-10, and IL-17A, and chemokines such as CXCL5, CXCL11, CXCL1, and CXCL6 distinguish MIS-C from paediatric patients with uncomplicated COVID-19. Studies have reported increased levels of TNF, IL-1β, IFNγ, soluble IL-2R, CCL2, CCL3, CCL4, CXCL8, or IFNγ-induced chemokines CXCL9 and CXCL10 in the serum of MIS-C patients compared to those with pCOVID-19 or healthy controls [5, 35, 48, 58, 71, 74–80]. Collectively, the enhancement of these pro-inflammatory molecules indicates inflammatory responses from myeloid and lymphoid cells.

### Innate immune responses in MIS-C

MIS-C is associated with dysregulated activation of innate immune cells, leading to widespread inflammation and tissue damage.

#### Neutrophils

Neutrophils, the most abundant circulating phagocytes, play a vital role in the body’s first line of defense [81]. However, excessive neutrophil hyperactivation can contribute to severe COVID-19 in adults [82, 83]. Compared to pCOVID-19, children with MIS-C have a significantly higher proportion of neutrophils in their blood [84]. Neutrophils in pCOVID-19 and MIS-C patients exhibit different characteristics. In pCOVID-19, acute infection induces an interferon-stimulated gene (ISG) signature in neutrophils, similar to the early infection stages in adults [82, 85], indicating an acute antiviral response. Additionally, pCOVID-19 neutrophils display an atypical phenotype with low expression of adhesion molecules (CD11b, CD66b, and L-selectin) and high expression of the inhibitory receptors (LAIR-1 and PD-1) and the activation markers (HLA-DR, CD64, and PECAM-1) [84]. This phenotype might prevent neutrophil infiltration into pulmonary capillaries, protecting against tissue injury in pCOVID-19. Consistent with these findings, serum or plasma from pCOVID-19 shows higher levels of IFN-α2a, associated with type I IFN response, and lower levels of IL-33, an epithelial and endothelial cell alarmin [63].

**Figure 2.**
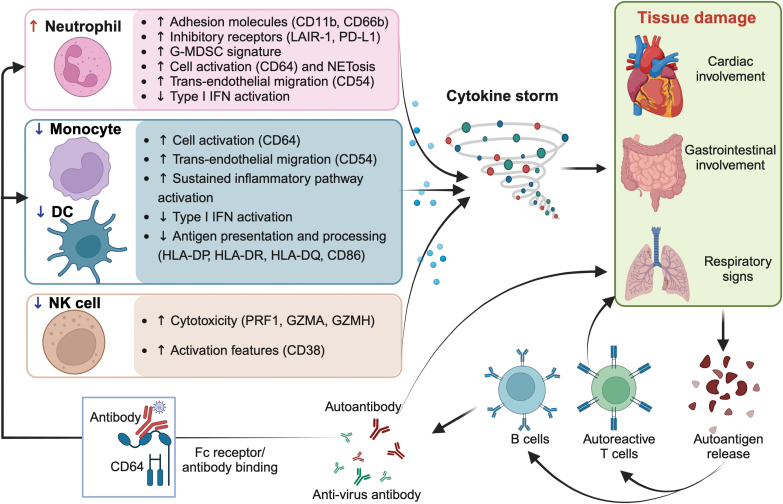
**The dysregulated innate immune response and autoimmunity.**In MIS-C, dysregulated activation of innate immune cells triggers hyperinflammation/cytokine storm, leading to tissue damage and the release of autoantigens. Repeated exposure to SARS-CoV-2 antigens, the release of autoantigens, and immune dysregulation may induce autoimmune disorders by activating autoreactive T cells and B cells. The induced antibody may bind the CD64 molecule on myeloid cells, which further triggers inflammation and tissue damage together with autoantigens and autoantibodies. This figure was created with BioRender.com

Neutrophils from MIS-C patients, on the other hand, exhibit higher expressions of CD11b, CD66b, LAIR-1, and PD-L1 compared to healthy controls [84] (Fig. 2). CD64, also known as Fc gamma receptor I (FcγRI), is a high-affinity receptor for the Fc region of IgG and a neutrophil activation marker, which can engage autoantibodies and immune complexes to trigger inflammation and tissue injury [86]. The expression of CD64 in neutrophils from MIS-C is notably increased compared to pCOVID-19 and healthy controls [48, 71, 84]. In MIS-C, extensive spontaneous neutrophil extracellular trap (NET) formation is observed, along with neutrophil activation and degranulation signatures. The SARS-CoV-2 antigen:antibody immune complexes (ICs) trigger NETosis, suggesting that the hyperinflammatory response observed in MIS-C could be mechanistically related to persistent SARS-CoV-2 antigenemia and driven by excessive neutrophil activation and NET release in the vasculature [85]. MIS-C neutrophils display a granulocytic myeloid-derived suppressor cell (G-MDSC) signature with highly altered metabolism [85], also suggesting dysregulated neutrophil activation and effector functions. The robust upregulation of CD54 (ICAM1) expression on neutrophils is observed in MIS-C individuals, indicative of APC activation and trans-endothelial migration. However, these cells lack signs of active type I interferon (IFN) signalling [48].

#### Monocytes and dendritic cells

Monocytes and dendritic cells (DC) are vital components of the innate immune system, responding to pathogens. In conditions such as viral infections, monocytes are activated and recruited by inflammatory mediators to affected tissues, where they differentiate into macrophages and DC-like phenotypes to perform pro- and anti-inflammatory activities, antigen presentation, and tissue remodelling [87]. DCs are unique antigen-presenting cells that induce primary immune responses and prime naïve T cells [88]. As reported, a decreased trend of circulating monocytes and DCs is observed in MIS-C patients [48, 63, 66, 75, 77, 89]. Similar to neutrophils, monocytes from MIS-C patients show elevated CD54 and CD64 expression, but no type I IFN signatures [48, 71] (Fig. 2). The upregulation of CD163 expression and several S100A family inflammatory genes are reported in MIS-C monocytes [63, 75]. However, classical monocytes from patients with MIS-C showed repressed inflammatory signatures compared to pCOVID-19 and paediatric healthy controls [63]. MIS-C patient monocytes/DCs reveal a significant reduction in antigen presentation and processing with reduced expression of HLA class II molecules (*HLA-DP, DQ*, and *DR*) and *CD86* [54, 71, 75]. Severe MIS-C results in myocarditis, predominantly affecting monocytes/DCs. In children with MIS-C and severe myocarditis, These cells show persistent nuclear factor κB (NF-κB) activation, increased TNF-α signalling, decreased expression of NF-κB inhibitors, and a hypoxic response driven by oxidative stress and VEGF signalling [89]. Elevated inflammatory cytokines (such as TNFα, TGFβ, IL1β, IL-13, IL-4, and VEGF) in MIS-C with severe myocarditis may mediate angiogenesis and vascular homeostasis and enhance cardiac fibroblasts’ development into cardiac myofibroblasts.

#### Natural killer cells

Natural killer (NK) cells are also part of the innate immune system with cytotoxicity and cytokine-producing effector functions [90]. Children with MIS-C exhibited decreased NK cells [48, 66, 74, 91, 92], but the role of NK cells in the pathogenesis of MIS-C remains elusive. Current evidence shows elevated cytotoxicity/activation features (PRF1, GZMA, GZMH, and CD38) with potential relevance for tissue damage in MIS-C NK cells [66, 75] (Fig. 2). There is a positive correlation between plasma IFNγ levels and natural cytotoxicity triggering receptor 1 (NCR1), the soluble marker of NK cells [93].

### T cell immune responses in MIS-C

T cells are a critical component of the immune system, categorized into αβ and γδ T cell subtypes based on their T cell receptors (TCRs). αβ T cells, the most common type, are prevalent in the blood and lymphoid organs, and include CD4^+^ and CD8^+^ T cells, playing a central role in adaptive immunity. γδ T cells, rare in lymphoid organs but more abundant in peripheral tissues like the skin, intestines, and lungs, exhibit both innate and adaptive immune characteristics, with antiviral properties such as IFN secretion, pathogen elimination, and cytotoxic activity [94]. Lymphopenia is common in MIS-C [6, 7, 59, 71, 74, 76, 95, 96]. Children with MIS-C display reduced T cell frequencies relative to healthy donors and encompass CD4^+^, CD8^+^, and γδ T cells [48, 59, 66, 71].

#### CD4^+^ T cells

Circulating CD4^+^ T cells tend to lower frequencies in MIS-C patients [48, 59, 66, 71]. The HLA-DR on T cells is a marker indicative of activation. In the early phase of MIS-C, the proportions of CCR7^+^CD4^+^ T cells (mainly naïve T cells and a small proportion of central memory T cells) increase, with more HLA-DR expression [71]. Other studies showed increased frequencies of HLA-DR^+^CD38^+^ CD4^+^T cells compared to pCOVID-19, which are the activated T cells responding to viral infection [66]. As reported by Hoste et al. [54], HLA-DR^+^CD38^+^CD4^+^T cells in MIS-C express high levels of TIM3, a marker for IFNγ-producing effector cells. Indeed, IFNγ levels correlate with TIM3 expression on HLA-DR^+^CD38^+^ T cells. In MIS-C, there is a high percentage of T cells, especially CD4^+^ T cells, expressing CCR6, which suggests trafficking to the endothelium, lungs, and gut [97]. In MIS-C, one potential mechanism of immune perturbation is chronic antigen exposure driving immune dysfunction or exhaustion [98]. In line with this hypothesis, more PD-1^+^CD4^+^ T cells are detected in MIS-C compared to pCOVID-19 [66]. Along with MIS-C recovery, there is a notable increase in regulatory T cell (Treg) counts [71].

#### CD8^+^ T cells

CD8^+^ T cells in MIS-C display marked activation and cytotoxicity features. Children with MIS-C have higher frequencies of HLA-DR^+^CD38^+^CD8^+^ T cells relative to pCOVID-19 [66]. Effector CD8^+^ T cells exhibit increased *PRF1*, *GZMA,* and *LAG3* expression when comparing severe MIS-C with paediatric healthy donors [75]. Increased frequency of PD-1^+^CD39^+^CD8^+^ T cells (exhausted) in MIS-C suggests a role for prolonged antigen stimulation in the inflammatory syndrome. CX3CR1 is the fractalkine receptor, which can adhere to fractalkine-expressing activated endothelium. The activation of vascular patrolling CX3CR1^+^CD8^+^ T cells is another distinguishing characteristic of MIS-C compared with pCOVID-19, especially in children requiring vasoactive medication [66]. This population may potentially contribute to the development of cardiovascular complications. Additionally, ITGB7, an integrin subunit supporting lymphocyte infiltration of the gut through MAdCAM-1 binding [99], was increased in memory CD8^+^ T cells from MIS-C individuals [75].

#### γδ T cells

The percentage of γδ T cells is decreased in the MIS-C relative to healthy donors, which returns to normal by convalescence. They also exhibit an increase in the HLA-DR expression [71].

#### TRBV11-2 and superantigen

**Figure 3.**
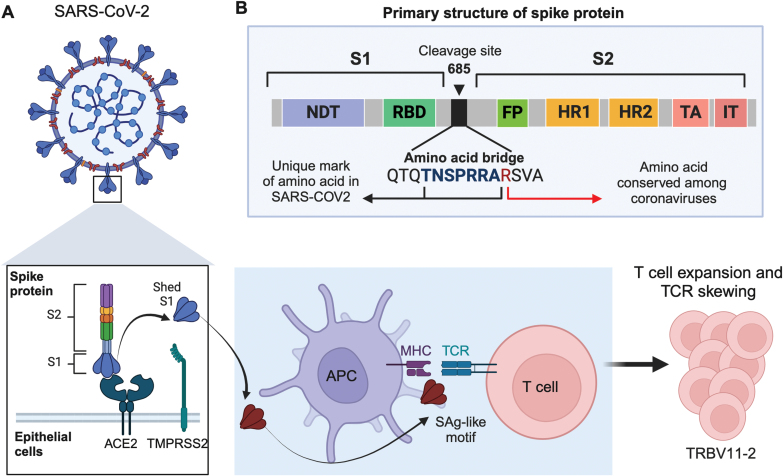
**The model of SARS-CoV-2 S protein as a SAg inducing T cell expansion and TCR skewing.**(A) SARS-CoV-2 S proteins on the virus surface interact with the host cell’s angiotensin-converting enzyme 2 (ACE2) receptor and transmembrane protease TMPRSS2. Upon binding to ACE2 receptors, the S proteins of SARS-CoV-2 are cleaved at the S1/S2 junction by proteases (TMPRSS2 and furin), facilitating membrane fusion and viral entry into the cell. This proteolytic cleavage occurs near the unique PRRA insert in SARS-CoV-2, located within the SAg-like motif adjacent to the S1/S2 cleavage site. This process separates the spike trimer into S1 and S2 subunits, forming the S2 fusion trimer, which remains attached to the viral membrane, and the S1 trimer, which is released into the extracellular space. The SAg-like motifs are exposed in the S1 subunit, and these motifs activate a large fraction of T cells, leading to TCR skewing. (B) Schematic of the primary structure of SARS-CoV-2 S protein [106]. The S protein comprises two functional subunits, S1 and S2, which are linked together by a polybasic amino acid bridge. The S1 subunit includes the N-terminal domain (NTD) and receptor binding domain (RBD). The S2 subunit contains the fusion peptide (FP), heptad repeat 1 and 2 (HR1 and HR2), transmembrane domain (TM), and intracellular tail (IT). The cleavage site between the S1 and S2 subunits is located at amino acid 685. This figure was created with BioRender.com

MIS-C shares clinical features with TSS, which is triggered by bacterial superantigens (SAgs). These SAgs activate T cells by binding to specific β chains of TCRs in a manner that is independent of the complementarity-determining region-3 (CDR3). As a result, SAgs can bypass TCR antigen specificity, inducing massive T cell activation and proliferation, which leads to a cytokine storm and potentially causes toxic shock [100–102]. The selective binding of SAgs to different TCR Vβ chains leads to Vβ skewing, where those T cells become overrepresented upon exposure to SAgs. Using structure-based computational models, Cheng et al. identified a high-affinity motif in the SARS-CoV-2 S glycoprotein for TCR binding [103]. The motif sequence is unique to SARS-CoV-2 and highly similar in both sequence and structure to the bacterial SAg staphylococcal enterotoxin B (Fig. 3A and 3B). They also reported TCR Vβ skewing in adult COVID-19 patients with severe hyperinflammation, suggesting SAg immune responses. Characterizing the TCR repertoire of MIS-C patients reveals a markedly increased frequency of *TRBV11-2* clonotypes [53–55, 63, 75, 79, 104]. But *TRBV11-2* TCRs do not pair with a specific TCRα, suggestive of superantigenic stimulation. Thus, it is hypothesized that SARS-CoV-2 protein, as a SAg, may directly mediate the extension of TRBV11-2. Indeed, polyacidic residues in the Vβ chain encoded by *TRBV11-2* strongly interact with SAg-like motif of SARS-CoV-2 S glycoprotein [53, 105].

The increased *TRBV11-2* usage is particularly notable soon after hospitalization, with a rapid decline observed thereafter [53, 63, 79, 104]. *TRBV11-2* T cells show more activated and cytotoxic phenotypes [54, 63]. In children with MIS-C, HLA class I alleles (A02, B35, and C04) are related to TRBV11-2, indicating that the interaction of T cells with HLA class I molecules on endothelial cells may drive tissue damage and autoantigen release [53, 63]. Activated T cells mediate cytokine storm. TRBV11-2 expansion correlates with levels of inflammatory cytokines, such as IL-6, IL-17, TNFα, and IFNγ [53, 55, 63].

### B cell immune responses and autoantibody

B cells participate in antigen presentation and are essential for humoral immune response, making them key players in defending against infections [107]. B cell-mediated responses are also aberrant in MIS-C. Acute MIS-Cs exhibit decreased frequencies of circulating B cells, which increases in the recovered cohort [71, 104]. The HLA-DR expression is notably reduced in B cells of the acute phase compared to healthy controls. In the resolution phase, more class-switched memory B cells and plasmablast cells are observed [71].

B cell-mediated humoral immune responses largely depend on the structural proteins of SARS-CoV-2. This virus contains two of the most important proteins: S glycoprotein which is the main antigenic target of cytotoxic lymphocytes and induces neutralized antibodies, and nucleocapsid (N) protein which is for viral RNA replication [5]. Adult COVID-19 cohorts have a broader antibody response of anti-S IgG, IgM, IgA, and anti-N IgG with increased neutralizing activity. This contradicts paediatric SARS-CoV-2 patients with predominant anti-S IgG and reduced neutralizing activity, independent of whether they develop MIS-C [50]. This indicates reduced age-dependent humoral response. Although strong antibody responses are detectable in most MIS-C patients, approximately 30% of them are PCR-negative for the virus [5, 52, 108]. Nevertheless, those antibody levels cannot distinguish PCR^+^ and PCR^−^ MIS-C patients [48]. Due to the delayed onset of MIS-C, IgM levels in MIS-C are lower compared to IgG, suggestive of prior SARS-CoV-2 exposure [47–52]. Bartsch et al. showed that children with MIS-C maintain highly inflammatory monocyte-activating SARS-CoV-2 IgG antibodies, distinguishable from children without MIS-C [52].

As pointed out above, neutrophils and monocytes from MIS-C upregulate CD64 (Fig. 2), a marker that can engage autoantibodies and immune complexes to trigger inflammation and tissue injury [86]. Gruber et al. identified IgG and IgA autoantibody repertoires against autoimmune disease-associated antigens (such as anti-La, a characteristic autoantigen of systemic lupus erythematosus and Sjogren’s disease), although these patients did not report autoimmune diseases before [48]. They also identified endothelial, gastrointestinal, and immune-cell antigens, which could partially explain the autoreactivity and immune dysregulation in MIS-C. In another study, Ramaswamy et al. provide evidence for endothelium-reactive IgG in clinically severe MIS-C patients [75]. The potential role of these autoantibodies in MIS-C pathogenesis needs to be further investigated. Notably, those cohorts were administrated by IVIG [48, 75]. It is unclear whether this administration may interfere with autoantibody detection and cause misleading results. Autoantibodies against IL-1R antagonists (IL-1Ra) or other autoimmune conditions, such as systemic lupus erythematosus, Sjögren’s syndrome, or autoimmune gastritis, were detected in more than half of MIS-C patients [48, 109, 110]. IgA levels are higher in MIS-C compared to pCOVID-19 patients [52]. However, IgA antibody titres are comparable between acute and convalescent phases of MIS-C. In concordance with elevated IgA titres and the gastrointestinal symptom of MIS-C, IL-17A activation and mucosal chemotaxis (CCL20 and CCL28) were noted [48].

### Treatment of MIS-C and immune alternation

The magnitude of the inflammatory response in MIS-C correlates with disease severity [58, 75], and treatment with glucocorticoids and IVIGs has been shown to improve clinical outcomes [62–65]. Following IVIG treatment, there is a decrease in biomarkers associated with type II IFN response (IFNγ and CXCL9), T cell activation (sCD25), cell adhesion (sE-Selectin/sCD62E), and monocyte/macrophage activation (sTNFRII, M-CSF, ferritin, and IL-6) [63]. Among patients treated with glucocorticoids, either alone or in combination with IVIG, a negative correlation was observed between the duration of hospitalization and the levels of most soluble biomarkers. The concurrent use of glucocorticoids with IVIG had a more specific effect on specific biomarkers, including IL-1Ra, MPO, sIL-2Rα, sTNFRI, LBP, sICAM-1, CCL3, and sCD163 [63]. Additionally, the elapsed time since the first administration of glucocorticoids showed a negative correlation with the frequency of TRBV11-2 clonotypes. This suggests that glucocorticoids may contribute to apoptosis transcriptional signatures observed in the single-cell analysis discussed above, potentially reflecting the contraction of CD4^+^ T cell subsets during disease resolution [63].

## Conclusions

The emergence of MIS-C as a severe complication in children following SARS-CoV-2 infection has highlighted the unique immune responses in paediatric patients. Despite its initial recognition as a condition similar to KD, MIS-C exhibits distinct epidemiological, clinical, and immunological profiles. The intense immune response and hyperinflammation seen in MIS-C contrast sharply with the typically milder impact of COVID-19 on children. Understanding the immune dysregulation in MIS-C is important. The innate and T/B cell-mediated immune mechanisms that may predispose certain paediatric subgroups to develop MIS-C or to experience mild or asymptomatic outcomes after SARS-CoV-2 infection are still being explored. Differences in immune profiles among patient cohorts may arise from variations in analytes measured, genetic and epigenetic backgrounds, disease severity, geographical location, and timing of analyses. Further research is required to address these variations and clarify the pathophysiological mechanisms of MIS-C. Reducing the existing heterogeneity in immunological studies will be essential to better understand and manage MIS-C.
